# Atypical Stevens-Johnson Syndrome in a Patient With Mycoplasma pneumoniae Infection: A Case Report

**DOI:** 10.7759/cureus.70669

**Published:** 2024-10-02

**Authors:** Sana Aleem, Ishrat Fatima, Sefal Mansoor, Muhammad Owais Khalil

**Affiliations:** 1 Acute Medicine, University Hospitals of Derby and Burton NHS Foundation Trust, Derby, GBR; 2 Internal Medicine, Queen's Hospital Burton, Burton, GBR; 3 General Practice, Health Education England East Midlands, Derby, GBR; 4 Internal Medicine, University Hospitals of Derby and Burton NHS Foundation Trust, Burton, GBR

**Keywords:** mycoplasma pneumoniae-associated mucositis, community acquired pneumonia, mycoplasma pneumoniae, fuchs syndrome, steven johnson syndrome

## Abstract

Stevens-Johnson syndrome (SJS) is a serious condition involving the skin and mucous membranes and is characterized by extensive necrosis and detachment of the epidermis. We present a case report of atypical SJS occurring as a complication of *Mycoplasma pneumoniae* infection in a young adult patient. This case report aims to add to the limited body of literature that exists on the topic and remind clinicians of the possible diagnosis of atypical SJS in the setting of mucosal rash associated with *M. pneumoniae* infection.

## Introduction

*Mycoplasma pneumoniae* is a short rod bacterium that primarily causes respiratory tract infections including pneumonia. It causes upper and lower respiratory tract infections in all age groups, particularly between five and 40 years of age. It is now considered one of the common causes of community-acquired atypical pneumonia. Extrapulmonary complications can be seen in approximately 25% of the patients diagnosed with *M. pneumoniae*. Extrapulmonary manifestations include encephalitis, meningoencephalitis, psychosis, autoimmune haemolytic anaemia, hepatitis, arthritis, pericarditis, endocarditis, myocarditis and atypical Stevens-Johnson syndrome (SJS) [1].

## Case presentation

**Figure 1:**
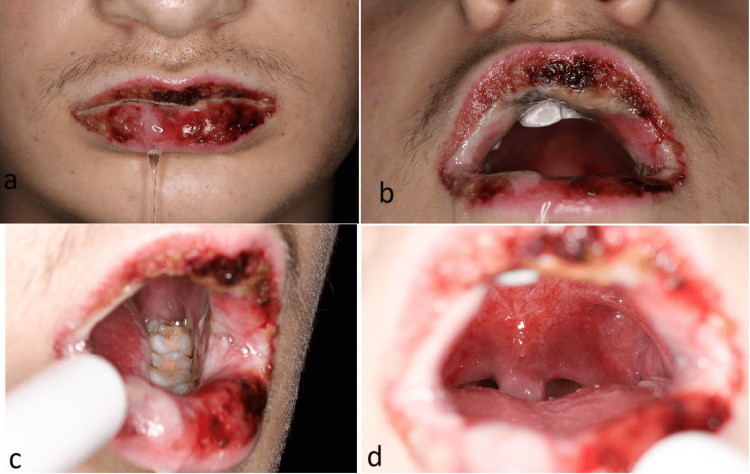
Lesions on the oral mucosa (a) Necrotic haemorrhagic crusting involving the lips. (b) Lesions involving the mucocutaneous junction. (c) Widespread erosion of the oral mucosa. (d) The pharynx showed diffuse erythema and white exudative lesions.

**Figure 2:**
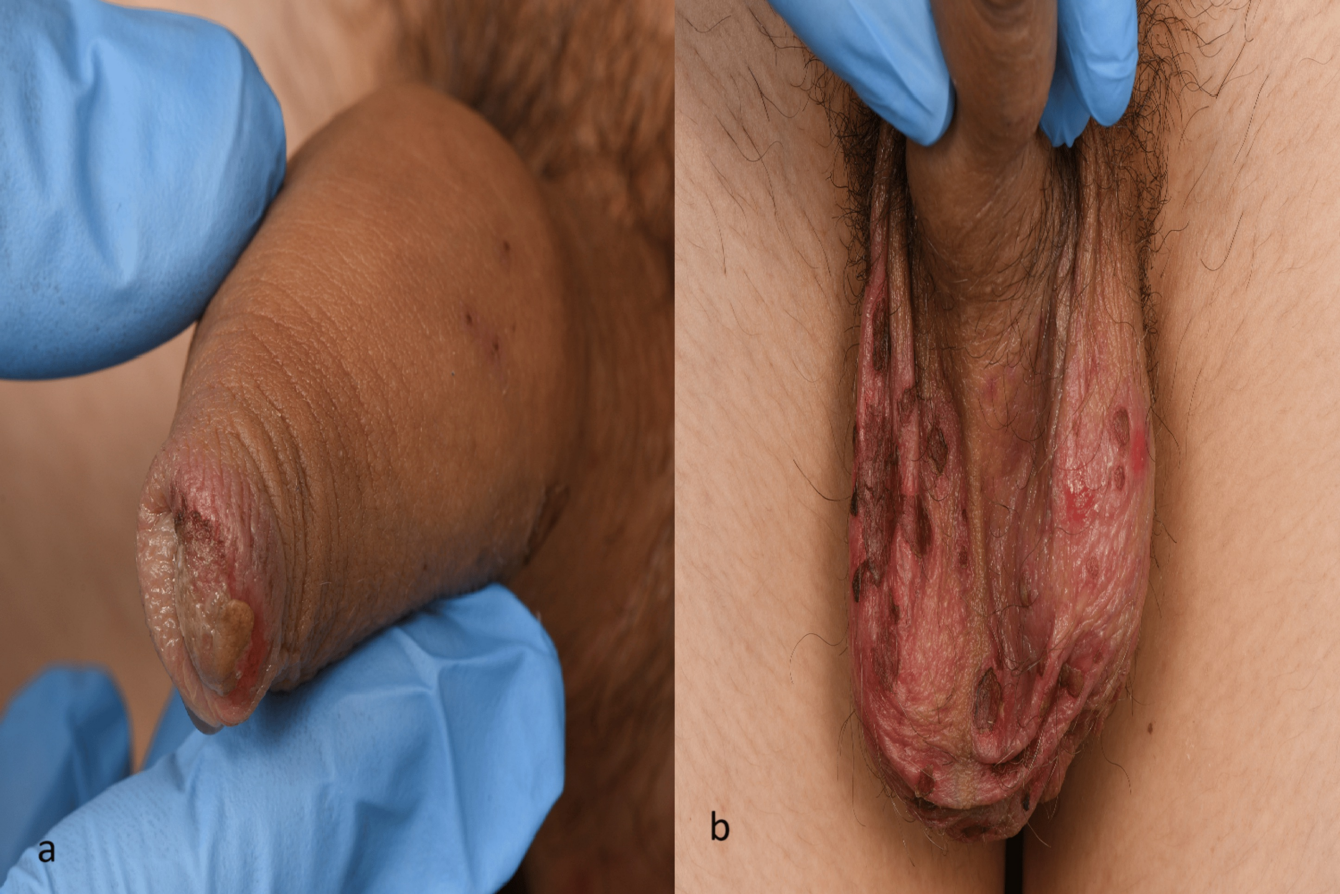
Lesions involving the genitals (a) A small ulcer with a necrotic base on the urethral meatus. (b) Small grouped vesicles on the scrotum.

A 16-year-old male with a past medical history of asthma presented with a 10-day history of cold-like symptoms including intermittent fever and productive cough to our hospital. He had been seen by his primary care physician five days ago and was prescribed oral amoxicillin for a presumed bacterial infection. No improvement was seen with the antibiotics. Six days into the illness, he developed painful ulcers in his mouth, on the lower lips (Figure 1) and on the genitals (Figure 2). He also noted a penile discharge one day prior to hospital admission. The patient denied any known drug allergies or history of sexual intercourse and recent travel. There was no family history of any drug allergies. His asthma had been well controlled on steroids and salbutamol inhalers. He was otherwise fit and active. Vital signs were as follows: respiratory rate 17 breaths per minute, oxygen saturation 96% on 1L supplemental oxygen and 91% without supplemental oxygen, blood pressure 120/70 mmHg, heart rate 76 beats/min, temperature 36.7°C (Table 1). Physical examination revealed widespread erosion of the oral mucosa with necrotic haemorrhagic crusting involving the labial mucosa and mucocutaneous junction. The mouth and pharynx showed diffuse erythema, ulcerations and white exudative lesions. A small ulcer with a necrotic base and sore was present on the urethral meatus, and small grouped vesicles on the scrotum were also noted. In addition, he demonstrated injected conjunctiva bilaterally without purulent discharge. There was no palpable lymphadenopathy, and no targetoid lesion or skin rash was observed. The rest of his examination was unremarkable.

**Table 1:** Laboratory results WBC: white blood cells; ALT: alanine aminotransferase; ALP: alkaline phosphatase; IgG: immunoglobulin G; IgA: immunoglobulin A; IgM: immunoglobulin M; ANCA: antineutrophil cytoplasmic antibodies; Anti-ENA: anti-extractable nuclear antigens; Anti-mitochondrial Ab: anti-mitochondrial antibody; Anti-smooth Muscle Ab: anti-smooth muscle antibody; Liver/Kidney Microsomes Ab: liver/kidney microsomes antibody; C3: complement component 3; C4: complement component 4

Investigations	On Admission	Before Discharge	Reference Range
WBC	20.8 (10^9^/L)	8.7 (10^9^/L)	4.0-10.0 (10^9^/L)
Haemoglobin	152 g/L	146 g/L	130-170 g/L
Platelet Count	212 (10^9^/L)	338 (10^9^/L)	150-410 (10^9^/L)
Neutrophils	16.7 (10^9^/L)	5.5 (10^9^/L)	2.0-7.0 (10^9^/L)
Lymphocytes	0.9 (10^9^/L)	1.9 (10^9^/L)	1.0-3.0 (10^9^/L)
Monocytes	3.0 (10^9^/L)	0.9 (10^9^/L)	0.2-1.0 (10^9^/L)
C-reactive Protein	16 mg/L	<5 mg/L	0-5 mg/L
Sodium	141 mmol/L	140 mmol/L	133-146 mmol/L
Potassium	3.1 mmol/L	3.5 mmol/L	3.5-5.3 mmol/L
Urea	5.0 mmol/L	5.0 mmol/L	2.5-7.8 mmol/L
Creatinine	61 umol/L	65 umol/L	59-104 umol/L
Calcium	2.35 mmol/mol	2.35 mmol/mol	2.20-2.60 mmol/mol
Phosphate	1.174 mmol/L	1.313 mmol/L	0.8-1.5 mmol/L
Magnesium	0.866 mmol/L	0.868 mmol/L	0.7-1.0 mmol/L
Total Bilirubin	7.0 umol/L	7.0 umol/L	0-21 umol/L
ALT	14 IU/L	61 IU/L	10-50 IU/L
ALP	99 IU/L	119 IU/L	30-130 IU/L
IgG	8.78 g/L	NA	5.49-15.84 g/L
IgA	2.06 g/L	NA	0.70-4.00 g/L
IgM	0.89	NA	-
Rheumatoid Factor	<10 KU/L	NA	0-14 KU/L
ANCA	Negative	NA	NA
Anti-ENA Screen	Negative	NA	NA
Anti-mitochondrial Ab	Negative	NA	NA
Anti-smooth Muscle Ab	Negative	NA	NA
Liver/Kid Microsomes AB	Negative	NA	NA
C3	1.370 g/L	NA	0.9-1.8 g/L
C4	0.35 g/L	NA	0.1-0.4 g/L
Connective Tissue Disease Interpretation	0.2	NA	NA

Suspicion for disseminated sexually transmitted illness most probably from a herpes simplex virus (HSV) infection was made, and the patient was started on acyclovir and antifungal medications. The infectious disease service was consulted, and they suspected an infection from Cytomegalovirus (CMV) or Epstein-Barr virus (EBV) and recommended continuing the current treatment.

We also consulted ENT, rheumatology and ophthalmology services. They suggested further testing, including an X-ray of the chest and viral serology. The chest X-ray showed bilateral, predominantly perihilar and medial patchy airspace with associated bronchial thickening, but there was no segmental or lobar consolidation. Lab investigations revealed negative viral serology for HSV, CMV, EBV, HIV, hepatitis B, hepatitis C, adenovirus, varicella-zoster virus and syphilis. Autoimmune and connective tissue disease screens were also negative. Despite this, the patient did not show significant improvement in symptoms. At this point, dermatology service was consulted, and they suspected either herpetic gingivostomatitis with genital involvement or a drug reaction secondary to amoxicillin, presenting as atypical SJS. They recommended obtaining a polymerase chain reaction (PCR) test for *M. pneumoniae* from the throat or nasal swabs, which came back positive. The diagnosis of *M. pneumoniae *complicated by atypical SJS was made, and the patient was started on IV clarithromycin 500 mg BD and a course of oral prednisolone at 1 mg/kg/day. He remained in the hospital for nine days, during which his mouth and genital ulcers began to heal. He was discharged home on oral antibiotics in stable condition.

## Discussion

SJS is a severe, life-threatening mucocutaneous reaction characterized by extensive blistering and skin detachment. It primarily affects skin and mucus membranes including eyes, mouth and genitals [2]. SJS without skin involvement is known as atypical SJS or *M. pneumoniae*-associated mucositis when caused by a Mycoplasma infection (also referred to as Fuchs syndrome). It is very rare and only a few cases have been reported so far [3]. *M. pneumoniae* is diagnosed by culture, serology and PCR. There is no single "gold standard" for the laboratory diagnosis of *M. pneumoniae*. However, serology is the most widely available investigation. Cultures take several weeks and have poor sensitivity. PCR is a very sensitive and rapid method to detect *M. pneumoniae* but may not be widely available. Macrolide antibiotics are the mainstay of the treatment for *M. pneumoniae* infection [4]. The diagnosis of SJS is mainly clinical, based on specific mucocutaneous lesions. In some cases, a skin biopsy can be obtained to confirm the diagnosis. The primary approach to managing SJS is to remove the trigger, such as by immediately discontinuing the causative drug, treating underlying infections, and providing supportive measures. Supportive care involves the management of fluids and electrolytes, wound care, and pain management. Patients with severe disease are managed in a specialized unit, such as an intensive care unit or burn care unit [2]. The use of systemic steroids remains controversial, with differing opinions and study outcomes contributing to ongoing debate. Some studies showed that the use of steroids can increase the risk of secondary infections, sepsis and prolonged hospital stays [5].

Drugs such as sulfa derivatives, nonsteroidal anti‐inflammatory agents, penicillin‐related and cephalosporin antibiotics, antiepileptics, allopurinol, and terbinafine are one of the common causes of SJS, especially in adults [2]. It is difficult to differentiate between drug-induced SJS and *M. pneumoniae*-associated SJS. However, *M. pneumoniae*-associated SJS can be seen more in young adults and the mucous membrane involvement is more pronounced than in drug-induced SJS. Moreover, respiratory symptoms tend to be seen more in *M. pneumoniae*-associated SJS as compared to drug-induced SJS [6].

## Conclusions

In the reported case, the lesions mainly involved the mucous membranes and conjunctiva without any skin involvement. Due to its unexpected presentation, the patient first visited the primary care physician and was started on amoxicillin for a presumed bacterial respiratory tract infection. In this case, although amoxicillin could be considered a potential trigger for SJS, considering the patient's age, clinical presentation, and predominant respiratory system involvement, it is more likely to be *M. pneumoniae*-associated SJS. The purpose of this case report is to remind the clinicians of the atypical SJS in the setting of *M. pneumoniae* infection and the need for prompt investigation and commencement of appropriate treatment.
